# GHITM regulates malignant phenotype and sensitivity to PD‐1 blockade of renal cancer cells via Notch signalling

**DOI:** 10.1111/jcmm.18290

**Published:** 2024-04-08

**Authors:** Shiyu Huang, Jiachen Liu, Juncheng Hu, Yanguang Hou, Min Hu, Banghua Zhang, Hongbo Luo, Shujie Fu, Yujie Chen, Xiuheng Liu, Zhiyuan Chen, Lei Wang

**Affiliations:** ^1^ Department of Urology Renmin Hospital of Wuhan University Wuhan Hubei China; ^2^ Institute of Urologic Disease, Renmin Hospital of Wuhan University Wuhan Hubei China; ^3^ Central Laboratory Renmin Hospital of Wuhan University Wuhan Hubei China; ^4^ Department of Cardiology Renmin Hospital of Wuhan University Wuhan Hubei China; ^5^ Hubei Key Laboratory of Digestive System Disease Wuhan China; ^6^ Department of Urology The Second Hospital of Huangshi Huangshi China

**Keywords:** GHITM, immunotherapy, malignant phenotype, Notch signalling, renal cancer

## Abstract

Growth hormone inducible transmembrane protein (GHITM), one member of Bax inhibitory protein‐like family, has been rarely studied, and the clinical importance and biological functions of GHITM in kidney renal clear cell carcinoma (KIRC) still remain unknown. In the present study, we found that GHITM was downregulated in KIRC. Aberrant GHITM downregulation related to clinicopathological feature and unfavourable prognosis of KIRC patients. GHITM overexpression inhibited KIRC cell proliferation, migration and invasion in vitro and in vivo. Mechanistically, GHITM overexpression could induce the downregulation of Notch1, which acts as an oncogene in KIRC. Overexpression of Notch1 effectively rescued the inhibitory effect induced by GHITM upregulation. More importantly, GHITM could regulate PD‐L1 protein abundance and ectopic overexpression of GHITM enhanced the antitumour efficiency of PD‐1 blockade in KIRC, which provided new insights into antitumour therapy. Furthermore, we also showed that YY1 could decrease GHITM level via binding to its promoter. Taken together, our study revealed that GHITM was a promising therapeutic target for KIRC, which could modulate malignant phenotype and sensitivity to PD‐1 blockade of renal cancer cells via Notch signalling pathway.

## 1 INTRODUCTION

Renal cell carcinoma (RCC), which accounts for 2% of malignancies, is the 16th most common cancer all over the world.^1^ With a prevalence of 70% among all RCC, KIRC remains the most common subtype of renal cancer.^2^ For low‐risk localized RCC, surgery is the first choice of the treatment, however, 20%–30% RCC patients are not diagnosed until the disease have developed into distant metastasis.^3^, ^4^ Although targeted therapy and immunotherapy have significantly improved the prognosis of metastatic renal cell carcinoma (mRCC), a median survival time of mRCC is only about 10 months.^5^, ^6^, ^7^ Hence, identifying effective biomarkers and therapeutic targets of RCC is urgently needed.

Li et al. first identified GHITM in a dwarf mouse line that expressed a growth hormone antagonist.^8^ Through phylogenetic analysis and bioinformatical tools, Kerstin Reimers et al summarized the molecular characteristic of GHITM and indicated it was one member of the Bax inhibitory protein‐like family.^9^ GHITM plays a significant role in maintaining mitochondrial homeostasis, Bruno Seitaj et al. reported GHITM could impact the mitochondrial protein synthesis machinery to sustain structure, shape and function of mitochondrial.^10^ However, up until now, the expression pattern and biological function of GHITM in tumour progression has not yet been reported.

A number of aspects of cancer biology are regulated by the Notch signalling pathway, including the conservation of cancer stem‐like cells, cancer immunity and angiogenesis.^11^ The dysfunction of Notch signalling is implicated in many cancers. Renal cell carcinoma tumorigenesis could be suppressed via inhibiting Notch signalling in vitro and in vivo.^12^ Nevertheless, the precise function of the four individual Notch receptors and the role of GHITM on Notch signalling pathway in KIRC remains elusive.

Recently, tumour immunotherapy, especially targeting the programmed death 1/programmed death‐ligand 1 (PD‐1/PDL1) pathway, have become the focus of research in KIRC.^13^, ^14^, ^15^ Moreover, tumour‐infiltrating immune cells are closely associated with prognosis and response to immunotherapy and play a pivotal role in the progression of KIRC.^16^, ^17^ The immunotherapy and targeted‐therapy of KIRC have evolved over the past three decades, but some important questions still remain regarding biomarkers of efficacy, patient selection and the optimal combination and sequencing of agents. Hence, it is of great significance to find a novel molecular target to guide KIRC therapy.

Yin Yang‐1 (YY1) is a widely expressed zinc finger transcription factor that exerts significant regulatory control over gene transcription in various cellular processes, encompassing cell proliferation, differentiation and tumorigenesis.^18^, ^19^ As a multifunctional protein, YY1 was previously shown to have dual roles in gene activation and repression depending on the cellular context,^20^ and it may be involved in the transcriptional regulation of 10% of the total mammalian gene set.^21^ Physiologically, YY1 is found to be overexpressed in several malignancies, including prostate cancer,^22^ colon cancer,^23^ metastatic breast cancer^24^ and gastric cancer.^25^ Nonetheless, the function of YY1 in KIRC is still unclear and needs to be further elucidated.

Our research investigated the correlation between GHITM expression and KIRC clinical characteristics, as well as the possible role of GHITM in KIRC patients' prognosis, diagnosis, infiltrating immune cell and drug sensitivity. In vitro and in vivo, dysregulation of Notch signalling and attenuated malignant phenotype had been seen in GHITM overexpressed (GHITM‐OE) KIRC cells. Notch1 overexpression abolished the ability of GHITM to regulate these effects. We also found that overexpression of GHITM enhanced the antitumour efficiency of sunitinib and PD‐1 blockade in KIRC. Further studies showed that YY1 could regulate GHITM gene transcription by binding to its gene promoter. These findings revealed that GHITM was a novel prognostic indicator and a target for antitumour therapy in KIRC.

## 2 MATERIALS AND METHODS

### 2.1 Publicly‐available databases analysis

Clinical information and RNA‐seq data of KIRC were acquired from The Cancer Genome Atlas (TCGA‐KIRC) dataset and three sets of microarrays used for validation were acquired from the Gene Expression Omnibus (GEO) database. Table S1 provides details about above datasets. UALCAN database was used to investigate GHITM protein level in KIRC^26^ and STRING database is used for protein–protein interaction network functional enrichment analysis.^27^ Gene expression profiling interactive analysis (GEPIA) database and Kaplan–Meier plotting database were used to assess the prognostic value of GHITM in KIRC.^28^, ^29^ The association of GHITM expression with tumour immune infiltration and relationship between immune infiltrates and clinical outcome in KIRC were investigated using TIMER2.0.^30^ By utilizing GSEA, the LinkedOmics database was used to investigate the Kyoto encyclopaedia of genes and genomes (KEGG) pathways of GHITM and its coexpression genes.^31^

### 2.2 Construction and validation of a predictive nomogram

To better predict KIRC patients' prognosis, a nomogram according to age, gender, stage, T stage, N stage, M stage, grade and GHITM expression was developed through the R package ‘rms’. In addition, calibration curve based on time was used to assess the accuracy of the predictive nomogram at 1, 3 and 5 years.

### 2.3 Gene set enrichment analysis and drug susceptibility analysis

GSE126964 with a functional gene set file (c2.cp.kegg. v7.4.symbols.gmt) was analysed by GSEA to obtain pathways enriched by GHITM. High‐risk group and low‐risk group were divided according to GHITM expression. Gene sets with nominal *p* value less than 0.05 and FDR less than 0.25 were considered of statistically significant. To determine clinic value of GHITM, in the TCGA‐KIRC cohort, the half‐maximal inhibitory concentration (IC50) of common anticancer drugs was calculated via ‘pRRophetic’ R package.

### 2.4 Cell culture and treatments

786‐O cells, A498 cells, 769‐P cells, Caki‐1 cells, HK‐2 cells, 293 T cells, HUVECs and Renca cells were purchased from the ATCC (American Type Culture Collection) which have been authenticated by STR profiling and regularly tested for mycoplasma contamination. In a humidified atmosphere of 5% CO_2_ maintained at 37°C, 786‐O cells, A498 cells, 769‐P cells, Caki‐1 cells, HUVECs and Renca cells were cultured in RPMI‐1640 medium (Invitrogen, USA). 293 T and HK‐2 cells were cultured in DMEM medium (Invitrogen, USA). All cell culture media contained 10% FBS (foetal bovine serum) (GIBCO, USA).

### 2.5 Plasmids and lentiviruses

pcDNA3.1‐Flag‐GHITM and pcDNA3.1‐Flag‐Notch1 plasmids were obtained from Sangon Biotech (Shanghai, China). The coding sequences of GHITM and Notch1 were cloned into lentiviral pCDH vector. The virus particles were produced by cotransfection of 293 T cells with recombinant lentivirus vectors and helper plasmids (pMD2.G and psPAX2) according to the lentivirus packaging protocol of Addgene. The overexpression lentivirus infected indicated cell lines in the presence of 8 μg/mL polybrene (Beyotime, Shanghai, China).

### 2.6 RNA isolation, quantitative real‐time PCR (qRT‐PCR) and chromatin immunoprecipitation (ChIP) analysis

Cellular RNA was extracted using the TRIzol reagent (Invitrogen, USA), and PrimeScript™ RT Reagent Kit (TaKaRa, Japan) was utilized to process reverse transcriptase reactions. We performed qRT‐PCR using the Roche LightCycler 480 detection system. Relative mRNA level of the gene was determined by 2 ^−ΔΔCt^ method and normalized to GAPDH. Table S2 listed the specific primer sequences. The commercial ChIP Assay Kit (Beyotime, Shanghai, China) was used to perform ChIP analysis, briefly, KIRC cells were cross‐linked with 1% formaldehyde solution and quenched with 125 mM glycine. DNA fragments ranging from 200 to 500 bp were obtained via sonication. Then, the lysate was immunoprecipitated with anti‐YY1 or IgG antibody for 16 h at 4°C. Immunoprecipitated DNA fragments were amplified by qRT‐PCR with the GHITM promoter primer as follow: 5′‐CTTATAGCTGCGGCGAGTGA‐3′ (forward) and 5′‐GACGCAACATCGAAAGGACG‐3′ (reverse). The primers for GAPDH, 5′‐TACTAGCGGTTTTACGGGCG‐3′ (forward) and 5′‐TCGAACAGGAGGAGCAGAGAGCGA‐3′ (reverse), were used as a negative control.

### 2.7 Protein extraction and Western blot

Cells were lysed using RIPA buffer, and BCA protein assay kit (Beyotime, Shanghai, China) was utilized to calculate the protein concentration. Then cellular proteins were subjected to SDS‐PAGE gels and transferred to polyvinylidene difluoride membrane (Millipore, USA). Blots were blocked with 5% nonfat milk for an hour, before incubating with primary antibodies overnight at 4°C followed with appropriate secondary antibodies for an additional an hour at room temperature. The protein band was detected by ChemiDoc™ XRS + system and Image J software were applied for determination of the protein abundance. Primary antibodies used in Western blot analyses included anti‐GHITM (Proteintech, China, 16,296‐1‐AP), anti‐YY1 (Proteintech, China, 22156‐1‐AP), anti‐PD‐L1 (Proteintech, China, 28076‐1‐AP), anti‐Notch1 (Proteintech, China, 20687‐1‐AP), anti‐Notch2 (Proteintech, China, 28580‐1‐AP), anti‐Notch3 (Proteintech, China, 55114‐1‐AP), anti‐Notch4 (Abcam, UK, ab184742) and anti‐GADPH (Proteintech, China, 10494‐1‐AP).

### 2.8 Patient tissues and immunohistochemistry (IHC)

Seven pairs of KIRC tissues were obtained from patients undergoing urological surgery in Renmin Hospital of Wuhan University. The privacy rights of human subjects always be observed. All human participants, human data and human tissue were obtained with informed consent, and the study was approved by ethics committee of Renmin Hospital of Wuhan University. All methods were carried out in accordance with the principles expressed in the Declaration of Helsinki. For IHC, the sections of tissue were routinely dewaxed, rehydrated and subjected to citrate buffer. After blocking with 3% hydrogen peroxide and 10% goat serum, the tissue sections were incubated with specific antibodies for Ki67 or Notch1 (1:100 dilution) overnight at 4°C, followed by incubating with secondary antibody for 1 h at 37°C. After being visualized with diaminobenzidine, the tissue sections were observed by two investigators under a microscope.

### 2.9 Cell proliferation assay

According to instructions, we seeded 786‐O and 769‐P cells post transfection into a 96‐well plate at the density of 2 × 10^3^ cells per well. 10 μl CCK‐8 solution (Beyotime, Shanghai, China) was added to each well at selected times and incubated for an additional 3 h at 37°C. Absorbance at 450 nm was analysed by PerkinElmer Microplate reader. For clone formation assay, cells were seeded into 6‐well plates with 1000 cells per well and cultured for around 2 weeks. After 4% paraformaldehyde fixation and 0.1% crystal violet staining, clone formation analysis was processed. For 5‐Ethynyl‐2′‐deoxyuridine (EdU) assay, the EdU kit (Click‐iT EdU‐594 Cell Proliferation Kit, Servicebio, Wuhan, China) was used. Briefly, EdU was incubated with KIRC cells and detected through the click reaction.

### 2.10 Cell migration and invasion assay

Transwell assays were carried out according to our previous studies.^16^ Briefly, 1 × 10^5^ KIRC cells were resuspended in 200 μL serum‐free medium and added into the upper chamber of 24‐well transwell plate, while the lower chamber was filled with 600 μL 1640‐medium containing 30% FBS. After 24 h, the cells passed through membrane were counted under a microscope and the result was analysed by Image J.

For wound healing assays, cells were seeded into 6‐well plate before being scratched with a 10 μL pipette tip when cells were almost confluent. Then we cultured cells in FBS‐free medium and obtained images at 0 and 24 h after wounding.

### 2.11 Tube formation assay

HUVECs were cultured with different 786‐O or 769‐P cell‐conditioned media (CM) as described after seeding into the 24‐well plates coated with Matrigel. After 6 h, tube formation was observed and imaged microscopically.

### 2.12 Animal experiments

All protocols were approved by ethics committee of Renmin Hospital of Wuhan University and were carried out in strict accordance with the recommendations of the Guide for the Care and Use of Laboratory Animals of the National Institutes of Health. For xenograft tumour model, 6‐week‐old BALB/c nude mice were randomly divided into two groups to, respectively, accepted subcutaneously injection for 5 × 10^6^ 786‐O EV (empty vector) or 786‐O GHITM‐OE (overexpression) cells. Tumour volumes were assessed every 3 days, and calculated using the formula (L × W^2^)/2. The mice were sacrificed after 4 weeks, then the tumours were collected for further analyses. For lung metastasis model, 786‐O cells (1 × 10^6^) were injected via tail veins. After 8 weeks, mice were euthanized and lung tissues were collected and subjected to haematoxylin and eosin staining.

To evaluate the role of GHITM in the efficacy of PD‐1 blockade in renal cancer in vivo, 5 × 10^6^ Renca EV or Renca GHITM‐OE cells were subcutaneously injected into the flank of 6‐week‐old BALB/c mice, respectively. After the tumour reached a size of approximately 50 mm^3^, mice were randomized into different groups and treated with anti‐PD‐1 (BioXcell, Clone RMP1‐14)/IgG (200 μg, i.p., given at days 0, 3 and 6). On day 18, mice were euthanized and tumours were collected.

### 2.13 Statistical analysis

All experiments were carried out at least three times. All data in this study were presented as mean ± SD and analysed by SPSS22.0 software, GraphPad8.0 software and R4.1.0 software. Unpaired Student's *t*‐test or paired *t*‐test was performed to evaluate the difference between two groups, whereas the differences among multiple groups were compared using one‐way analysis of variance and further Tukey's post hoc test. Difference in clinicopathological factors between the high‐ and low‐GHITM expression groups was compared by a chi‐square test. The prognostic significance of genes was determined by univariate‐ and multivariate Cox regression analyses and Kaplan–Meier survival analysis. ROC curves were utilized to evaluate the diagnostic value of GHITM. *p* value less than 0.05 were considered statistically significant.

## 3 RESULTS

### 3.1 GHITM is downregulated in KIRC and correlated with clinicopathological features

**FIGURE 1:**
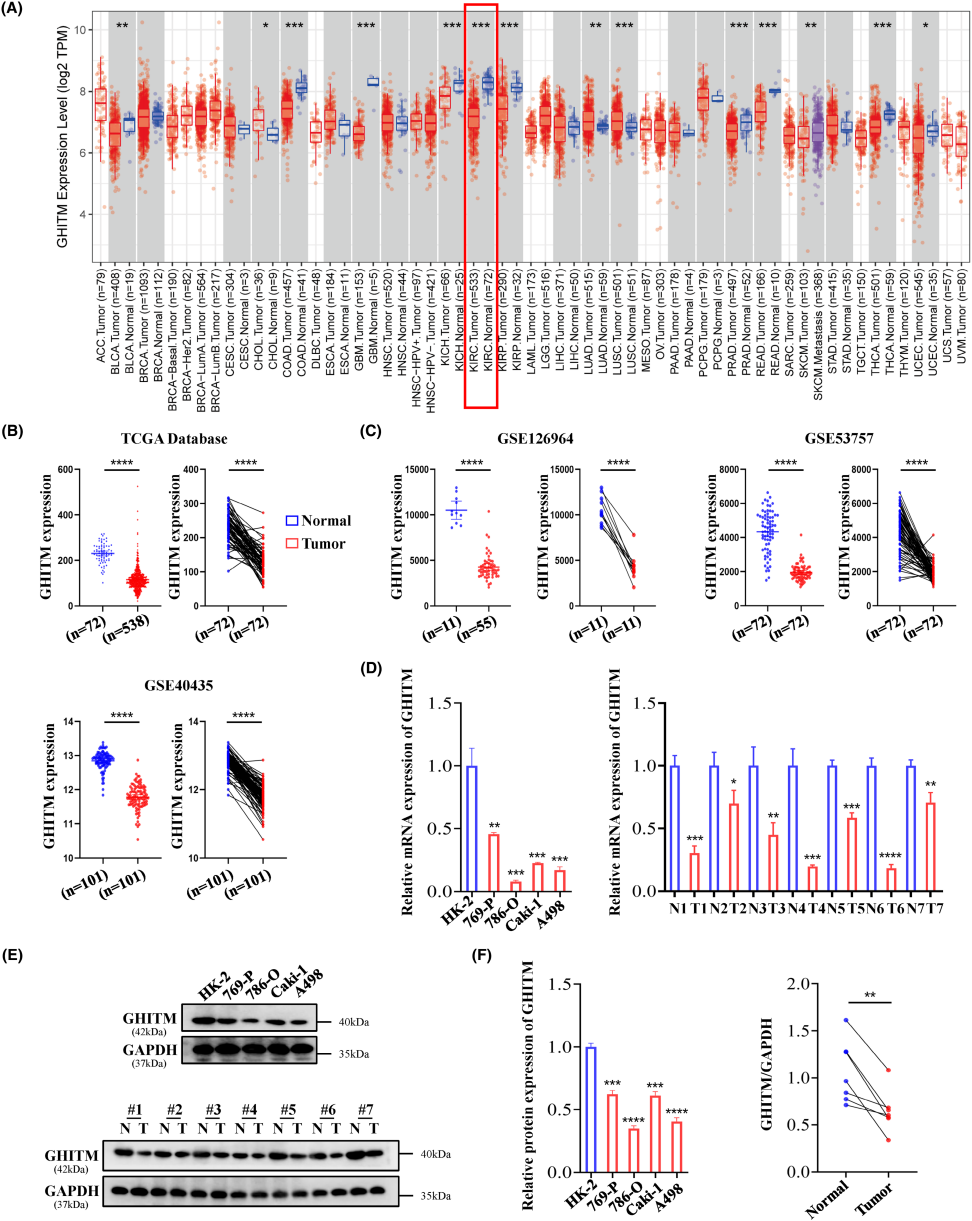
GHITM is downregulated in KIRC and correlated with clinicopathological features. (A) GHITM expression levels in different tumour types were determined by TIMER2.0 database. (B) GHITM expression in KIRC tumour and normal samples from TCGA database. (C) GHITM expression analysis in indicated GEO datasets (GSE126964, GSE53757 and GSE40435). (D) Analysis of GHITM mRNA levels in KIRC cell lines and seven paired KIRC specimens through qRT‐PCR. (E, F) Western blots and quantitative results. *p < 0.05; **p < 0.01; ***p < 0.001; ****p < 0.0001.

Few studies have been conducted recently on expression pattern and prognostic significance of GHITM in cancer. In our research, data from TIMER2.0 and GEPIA revealed that the low expression of GHITM was detected in KIRC (Figure 1A and Figure S1). TCGA samples and GEO series exhibited that compared with normal tissues, KIRC tumour tissues had a lower level of GHITM (Figure 1B,C). GHITM was downregulated in all variables in TCGA‐KIRC samples, including patient age, gender, stage of cancer and tumour grade, compared to normal. (Figure S2A). Consistent with the above‐mentioned bioinformatics analysis, the results of qRT‐PCR and Western blotting showed that GHITM mRNA and protein levels significantly downregulated in KIRC cell lines and KIRC tissues compared to normal kidney tubular epithelial cell HK‐2 and adjacent nontumour tissues (Figure 1D–F). Additionally, GHITM downregulation correlated closely with tumour stage and tumour grade in TCGA‐KIRC cohort (Table S3), lower GHITM expression was observed in advanced KIRC (Figure S2B). Table S4 displayed low level of GHITM was closely related to tumour grade in GSE40435 as well.

### 3.2 Diagnostic and prognostic value of GHITM in KIRC

**FIGURE 2:**
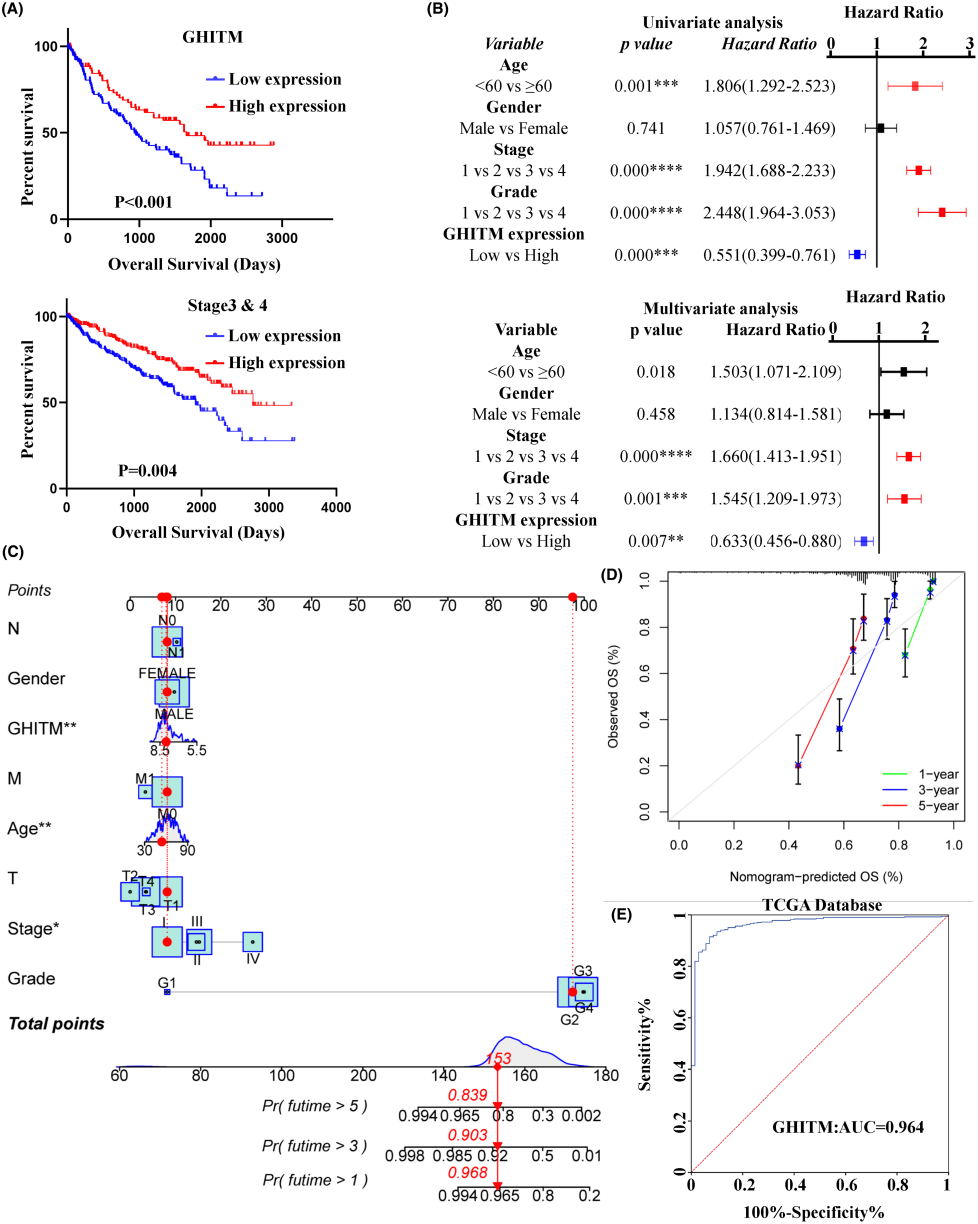
Diagnostic and prognostic value of GHITM in KIRC. (A) Analysis of the prognostic value of GHITM in TCGA‐KIRC cohort. (B) Univariate and multivariate analyses demonstrated GHITM expression as an independent predictor for OS of KIRC patients. (C) Nomogram for prediction of the 1‐, 3‐ or 5‐year survival rates for KIRC individuals. (D) Calibration curve analysis of the 1, 3 and 5 years. (E) ROC curves for KIRC patients in TCGA database. *p < 0.05; **p < 0.01; ***p < 0.001; ****p < 0.0001.

To determine the clinical significance of GHITM, we first explored the correlation of GHITM expression with the outcome of KIRC patients. As shown in Figure 2A and Figure S3A, the patients with low GHITM expression had worse outcomes than the patients with high GHITM expression. Assessment of prognostic significance of GHITM expression in KIRC patients subgrouped by age, gender, stage and grade also indicated low GHITM expression was related to poor prognosis in most of these groups (Figure 2A and Figure S3B). Univariate analysis and subsequent multivariate analysis displayed that GHITM expression was an independent risk factor associated with OS (Figure 2B). For better prognosis prediction, a nomogram based on GHITM expression for KIRC was constructed to predict the 1‐, 3‐ and 5‐year survival rates of patients, and there was a good agreement between prediction and observation according to corresponding calibration curves (Figure 2C,D). A 5‐year survival rate of early RCC is over 90%, but no efficient biomarker was found for early diagnosis of KIRC over the past several decades.^32^ GHITM diagnostic value in KIRC was evaluated using GEO and TCGA datasets. In TCGA‐KIRC, GHITM showed significant diagnostic accuracy with AUC = 0.964 (Figure 2E). To further explore the diagnostic significance of GHITM in early stages of KIRC, we separated the stage I patients and analysed the diagnostic value of GHITM. Figure S4 displayed that a high diagnostic value of GHITM was confirmed in GSE53757 with AUC = 0.968 and TCGA‐KIRC cohort with AUC = 0.956. In conclusion, our study indicated that GHITM might be a potential early diagnostic and prognostic biomarker for KIRC.

### 3.3 Upregulation of GHITM suppressed the malignant phenotype of KIRC cells in vitro and in vivo

**FIGURE 3:**
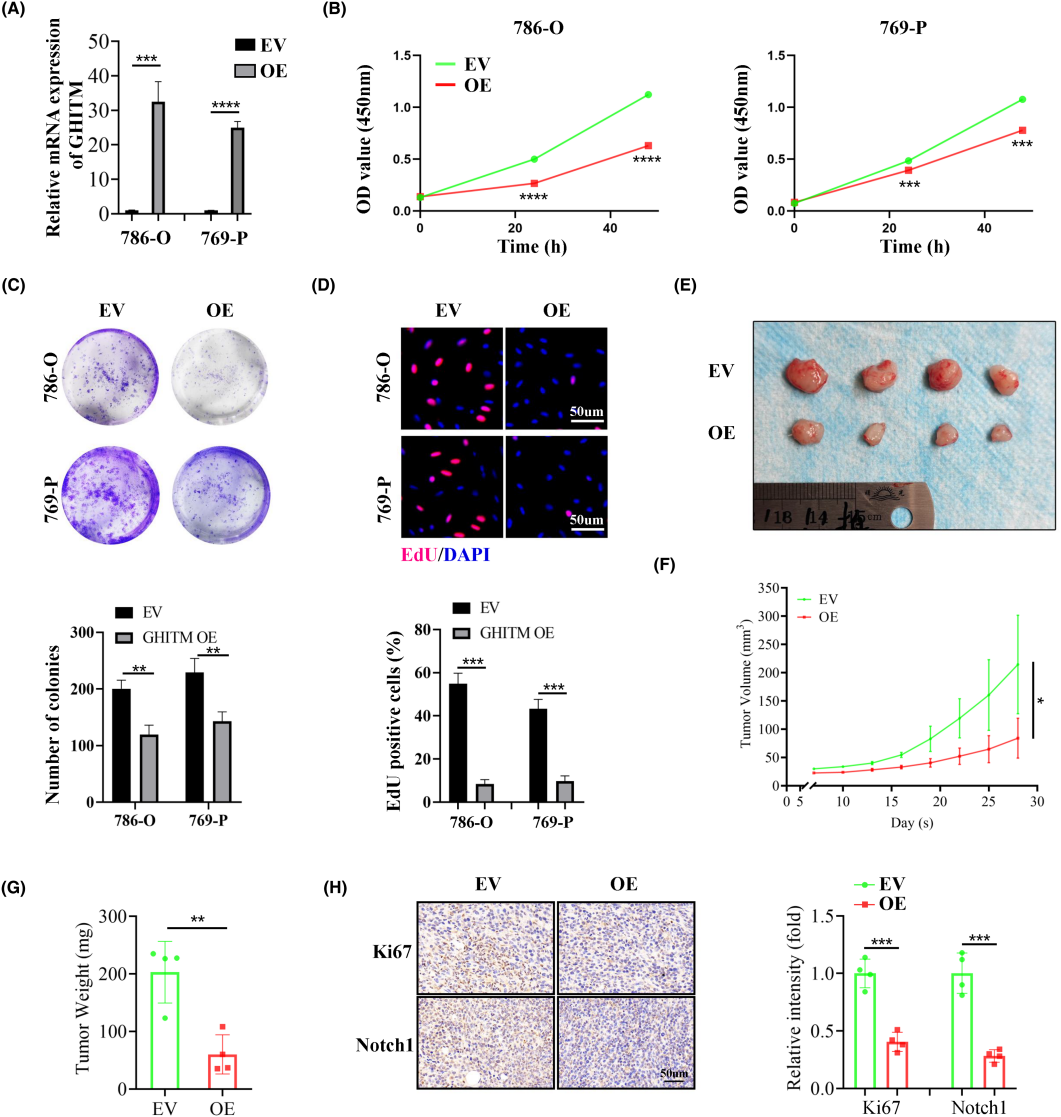
GHITM inhibits the malignant phenotype of KIRC cells in vitro and in vivo. (A) qRT‐PCR analysis of cells with GHITM overexpression. GHITM overexpression inhibited proliferation of KIRC cells, as demonstrated by (B) CCK‐8 assay, (C) clone formation assay and (D) EdU assay. (E–G) Representative images of the tumour, and the statistical results of tumour volume and weight. (H) Representative images and the quantitative results of IHC staining of Ki‐67 and Notch1 in tumour tissues. *p < 0.05; **p < 0.01; ***p < 0.001; ****p < 0.0001.

**FIGURE 4:**
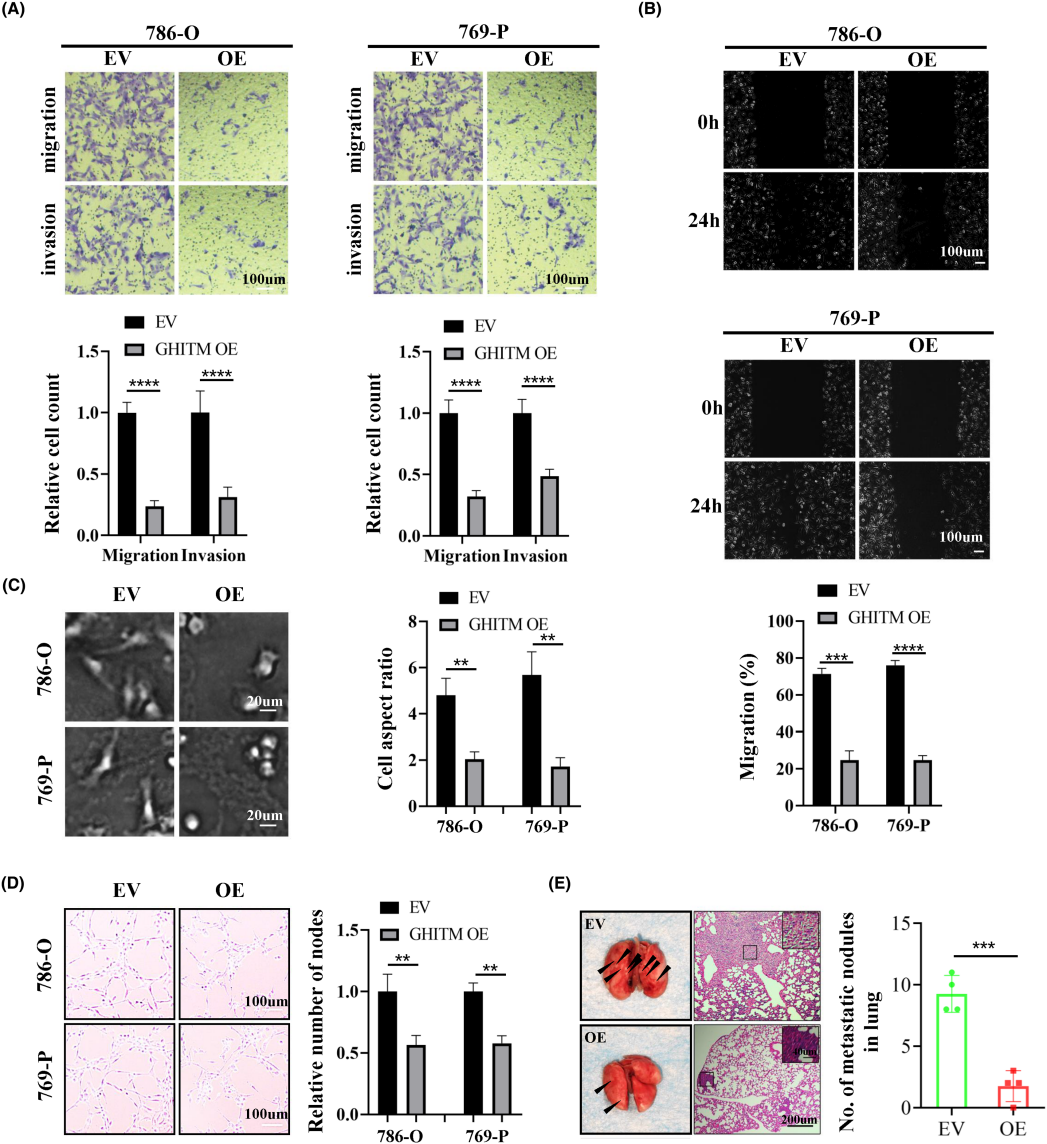
GHITM inhibits the malignant phenotype of KIRC cells in vitro and in vivo. GHITM overexpression attenuated the migration and invasion ability of KIRC cells indicated by transwell assay (A) and wound healing assay (B). (C) Representative images of EV and GHITM OE KIRC cells. (D) Tube formation assay in HUVECs cultured with medium collected from EV or GHITM OE KIRC cells. (E) Representative images and statistical data of lung metastases in mice. **p < 0.01; ***p < 0.001; ****p < 0.0001.

To investigate the function of GHITM in KIRC progression, 786‐O and 769‐P cells with GHITM stably overexpressed were established (Figure 3A). The results of CCK‐8, clone formation and EdU assays suggested GHITM overexpression decreased the proliferation of KIRC cells (Figure 3B–D). In line with the results of in vitro experiments, xenografts in the GHITM OE group were markedly smaller and less weighed than those in the GHITM EV group (Figure 3E–G). IHC staining of the xenografts demonstrated that Ki‐67 and Notch1 protein levels decreased in GHITM OE group than that in the control group (Figure 3H). Furthermore, wound healing assay and transwell assay showed GHITM upregulation could decrease the migration and invasion ability of KIRC cells (Figure 4A,B). Additionally, GHITM OE also induced a flat, cuboidal morphology of KIRC cells and abrogated conditioned medium‐induced tube formation (Figure 4C,D). As for pulmonary metastasis, 786‐O cells stably transfected with GHITM OE or GHITM EV lentiviruses were injected intravenously by tail vein for approximately 2 months. The metastatic lesions of the lungs in GHITM OE group were significantly reduced in comparison with those in the control group (Figure 4E).

Maria Patron et al. have demonstrated that GHITM could limit mitochondrial hyperpolarization and ROS production of cells,^33^ some studies also revealed the correlation between GHITM and cell apoptosis.^34^ Thus, whether GHITM could regulate ROS production and apoptosis of KIRC cells is worthy of researching further. Nevertheless, the results showed GHITM overexpression could not attenuate ROS generation or proportion of apoptotic cells of KIRC cell lines (Figure S5).

### 3.4 GHITM inhibits the malignant phenotype of KIRC cells through regulating Notch1

**FIGURE 5:**
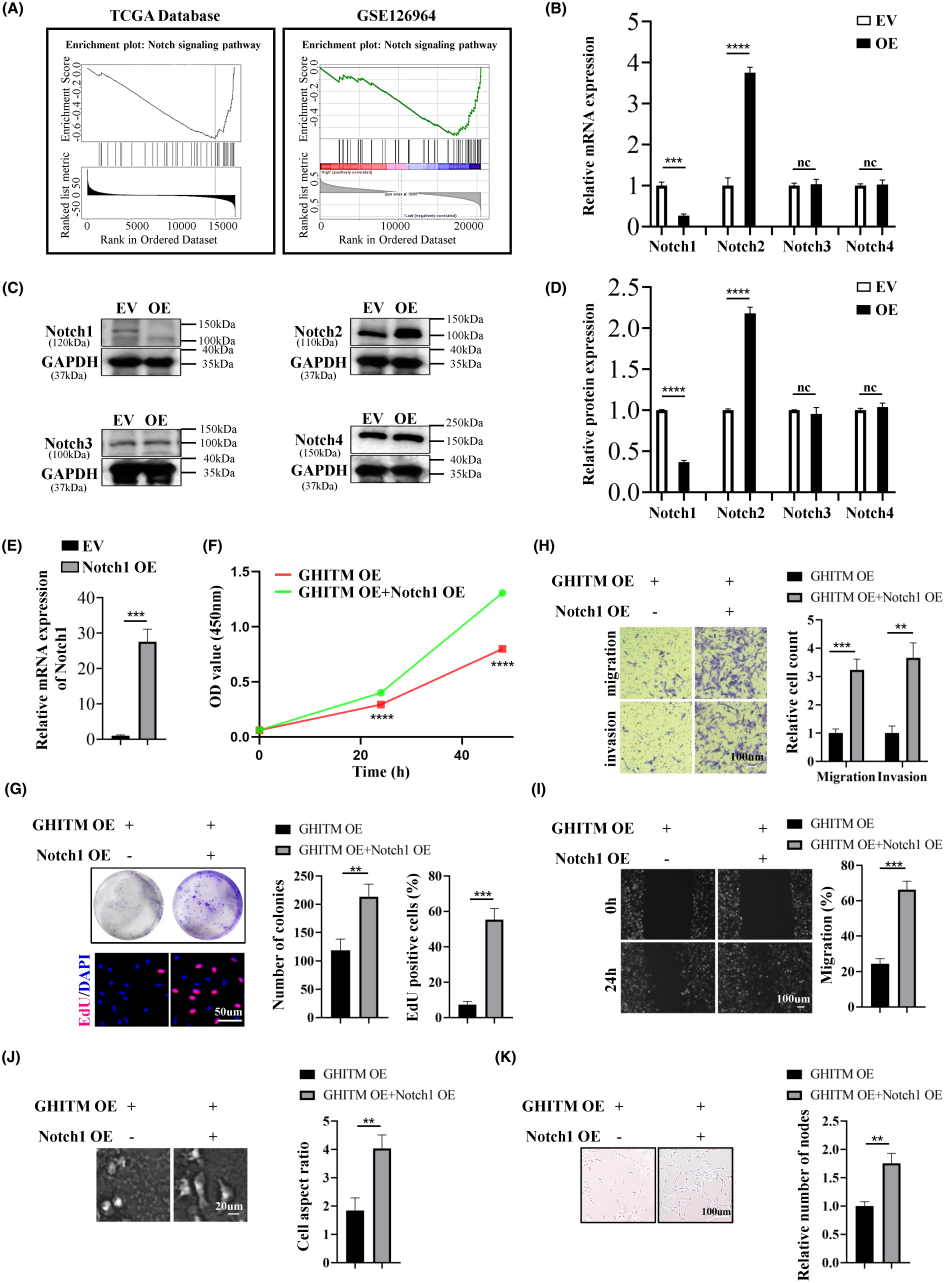
GHITM inhibits the malignant phenotype of KIRC cells through regulating Notch1. (A) GSEA analysis of the TCGA‐KIRC dataset and GSE126964. (B) qRT‐PCR analysis of indicated Notch1‐4 expression in GHITM overexpressed 786‐O cells. (C, D) Notch1‐4 protein expression and their quantitative analysis in indicated groups. (E) qRT‐PCR analysis of cells with Notch1 overexpression. (F) CCK‐8 assay for cell viability. (G) Representative images and quantitative results of clone formation and EdU assays. (H) Representative images and quantitative results of transwell assays. (I) Representative images and quantitative results of wound healing assay. (J) Representative images of 786‐O cells infected with indicated lentivirus. (K) Representative images and the quantitative results of tube formation assay. **p < 0.01; ***p < 0.001; ****p < 0.0001.

Notch signalling cascade is active in KIRC cell line and disturbing Notch signalling lead to reduction of proliferation of KIRC cells.^12^ Based on the LinkedOmics database, KEGG pathways of genes adversely associated with GHITM in KIRC were enriched, and the results of GSEA illuminated that the negatively correlated genes of GHITM in KIRC might partake in Notch signalling pathway (Figure 5A). GSEA based on GSE126964 dataset was also showed Notch signalling pathway that ranked the highest enrichment score was enriched in low‐GHITM level group (Figure 5A). qRT‐PCR and Western blotting analysis showed GHITM overexpression led to downregulation of Notch1 and upregulation of Notch2, while the expression of Notch3 and Notch4 had no change in the process (Figure 5B–D). Jonas Sjölund et al. concluded the growth‐promoting role of Notch signalling in KIRC cells was specifically attributed to the Notch1 receptor, in all KIRC cell lines tested, si‐Notch1 resulted in remarkable reductions in cell proliferation compared with control siRNA.^12^ Therefore, we overexpressed Notch1 in 786‐O GHITM OE cells and the results revealed that Notch1 OE abolished the inhibitory effects of GHITM on malignant phenotype of KIRC cells (Figure 5E–K). Collectively, these findings demonstrated that GHITM inhibited proliferation, migration and invasion of KIRC cells via Notch1.

### 3.5 GHITM expression correlated with tumour immune infiltrates (TILs) and drug sensitivity

**FIGURE 6:**
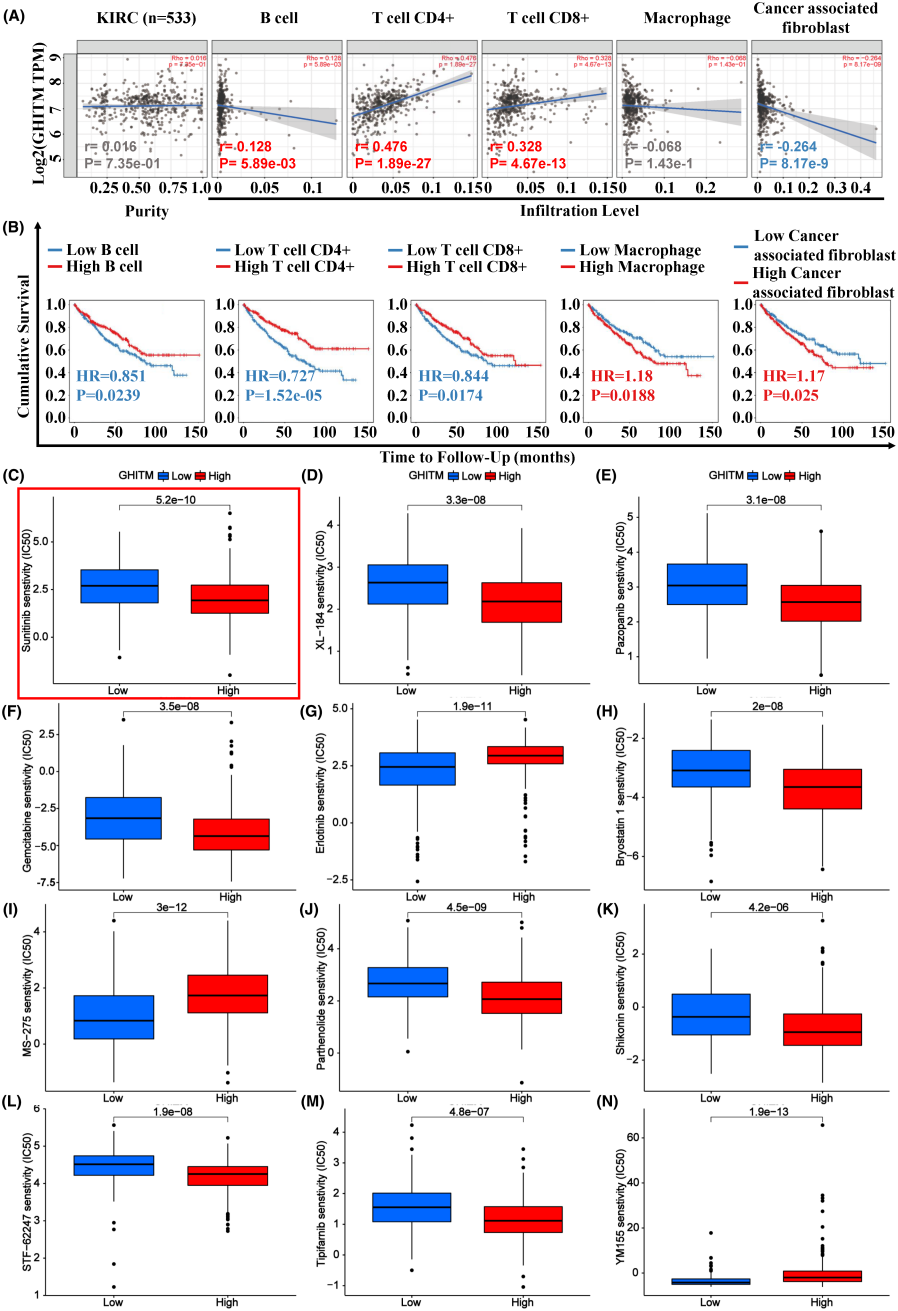
GHITM expression correlated with tumour immune infiltrates (TILs) and drug sensitivity. (A) Association between GHITM level and TILs in KIRC. (B) Kaplan–Meier survival analysis based on immune cells infiltration levels of KIRC patients. IC50 levels of indicated agents of KIRC, including (C) Sunitinib, (D) XL‐184, (E) Pazopanib, (F) Gemcitabine, (G) Erlotinib, (H) Bryostatin 1, (I) MS‐275, (J) Parthenolide, (K) Shikonin, (L) STF‐62247, (M) Tipifarnib and (N) YM155. HR, hazard ratio.

In the treatment of kidney cancer, tumour immunotherapy plays an important role and have attracted much attention in recent years. To explore the relationship of tumour‐infiltrating lymphocytes and GHITM level in KIRC, EPIC algorithm, a method for analysing bulk tumour gene expression data to calculate the proportion of immune cells and cancer cells, was conducted in TIMER2.0. The results revealed GHITM levels were positively correlated with antitumor immune cells infiltration levels including B cell (*r* = 0.128, *p* = 5.89e‐03), CD4+ T cell (*r* = 0.476, *p* = 1.89e‐27) and CD8 + T cell (*r* = 0.328, *p* = 4.67e‐13) (Figure 6A). The infiltration level of cancer associated fibroblast cells, the protumor immune cells, was negatively associated with GHITM levels (*r* = −0.264, *p* = 8.17e‐9) (Figure 6A). By Kaplan–Meier survival analysis, high infiltration level of B cell, CD4+ T cell and CD8+ T cell that positively correlated with GHITM level predicted better OS of KIRC patients, but high infiltration level of cancer associated fibroblast cells which negatively related to GHITM level predicted poor OS of KIRC patients (Figure 6B). Next, we investigated the relationship between GHITM expression and IC50 of anticancer drugs, including the drugs that were clinically proven to be effective in KIRC patients (Sunitinib, XL‐184, Pazopanib, Gemcitabine, Erlotinib and Bryostatin 1) and the drugs that were validated to be effective in the laboratory only (MS‐275,^35^ Parthenolide,^36^ Shikonin,^37^ STF‐62247,^38^ Tipifarnib^39^ and YM155^40^). As shown in Figure 6C–N, IC50 levels of Sunitinib, XL‐184, Pazopanib, Gemcitabine, Bryostatin 1, Parthenolide, Shikonin, STF‐62247 and Tipifarnib were higher in the low‐GHITM level group, suggesting high‐GHITM level KIRC patients were more sensitive to the indicated drugs. Whereas, IC50 levels of Erlotinib, MS‐275 and YM155 were higher in the high‐GHITM level group, suggesting low‐GHITM level KIRC patients were more sensitive to the indicated drugs. Moreover, we explored the association between GHITM expression of TCGA‐KIRC patients and their sensitivity to chemotherapeutic agents which were validated to be effective in other kinds of cancer (Figure S6A,B). These results implied the possible significance of GHITM in treatment of KIRC patients.

### 3.6 GHITM regulates sensitivity to sunitinib and PD‐1 blockade in KIRC

**FIGURE 7:**
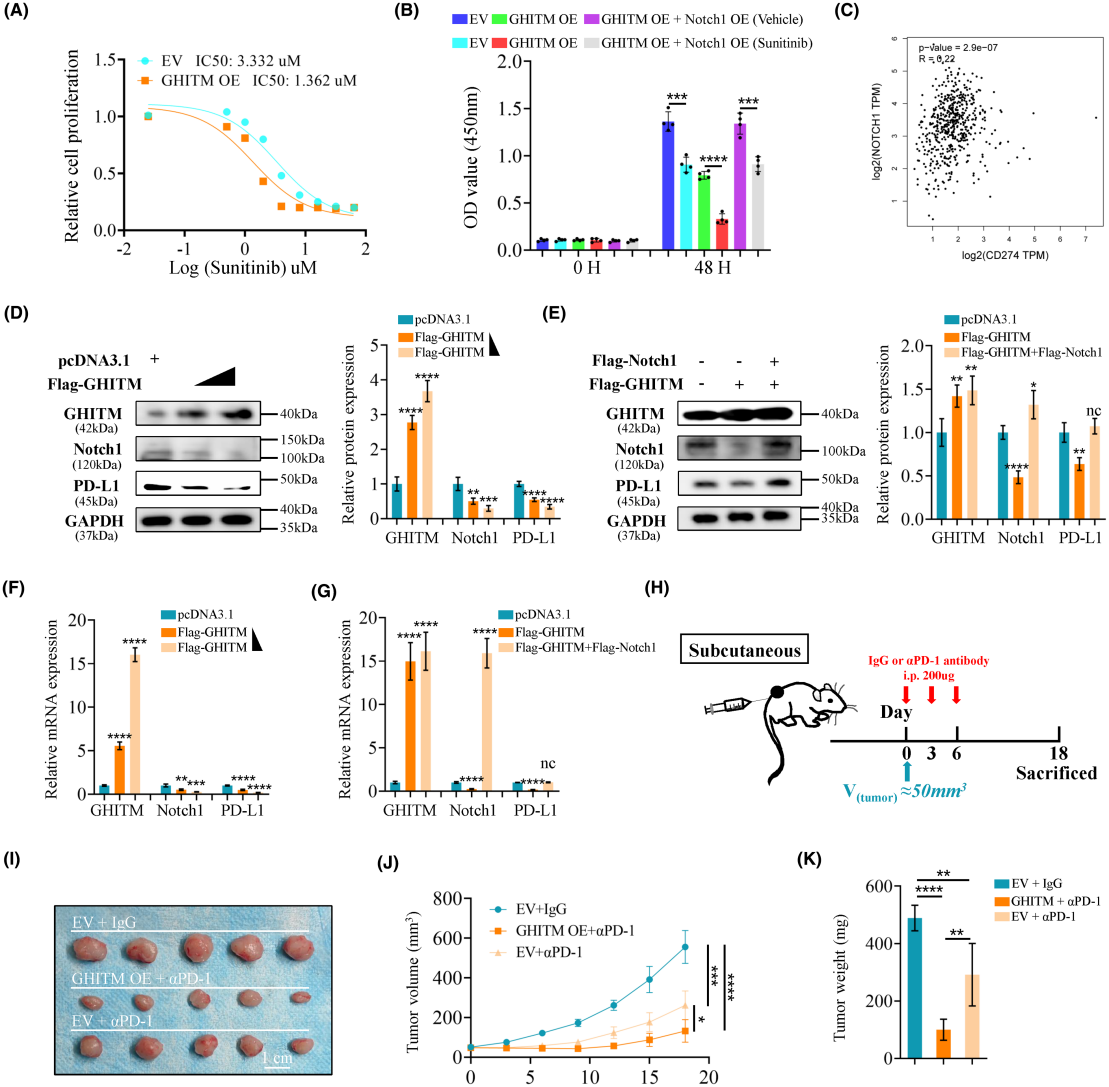
GHITM regulates sensitivity to sunitinib and PD‐1 blockade in KIRC. (A) 786‐O cells were treated with a series of concentrations of sunitinib for 24 h and harvested for CCK‐8 assay. (B) CCK8 assay for cell viability. (C) Scatterplots of correlations between Notch1 expression and PD‐L1 based on GEPIA database. (D, E) Western blots and quantitative results. (F, G) Relative mRNA levels of GHITM, Notch1 and PD‐L1 in indicated groups. (H) A schematic treatment plan for mice with subcutaneous Renca tumours. Tumour images (I), tumour volumes (J), and tumour weights (K) are shown. *p < 0.05; **p < 0.01; ***p < 0.001; ****p < 0.0001.

Tyrosine kinase inhibitors (TKIs) and immune checkpoint inhibitors improved overall survival in advanced renal cell carcinoma; however, many patients would develop resistance to targeted therapy and immunotherapy.^41^, ^42^ Our results showed that ectopic overexpression of GHITM reduced the IC50 value of sunitinib in 786‐O cells (Figure 7A). The CCK‐8 assay also demonstrated that overexpression of GHITM enhance the sensitivity of KIRC cells to sunitinib, which was consistent with previous bioinformatics analysis, and subsequent studies indicated that overexpression of Notch1 abolished the effect of GHITM on regulating the sensitivity of KIRC cells to sunitinib (Figure 7B).

Recently, results from Maurizio et al. suggested that Notch signalling could facilitate immune‐escape by upregulating PD‐L1.^43^ Intriguingly, GEPIA database also indicated that Notch1 level significantly correlated PD‐L1 level in KIRC (Figure 7C). To explore the role of GHITM in PD‐L1 expression in KIRC, we evaluated PD‐L1 levels after GHITM overexpression. As shown in Figure 7D–G, GHITM overexpression decreased the mRNA and protein levels of PD‐L1 in 786‐O cells, whereas, Notch1 overexpression prevented GHITM‐induced PD‐L1 downregulation. Importantly, GHITM overexpression could enhance the antitumor effects of PD‐1 blockade in BALB/c mice which were immunocompetent (Figure 7H–K).

### 3.7 YY1 regulates the expression of GHITM via binding to its promoter in KIRC

**FIGURE 8:**
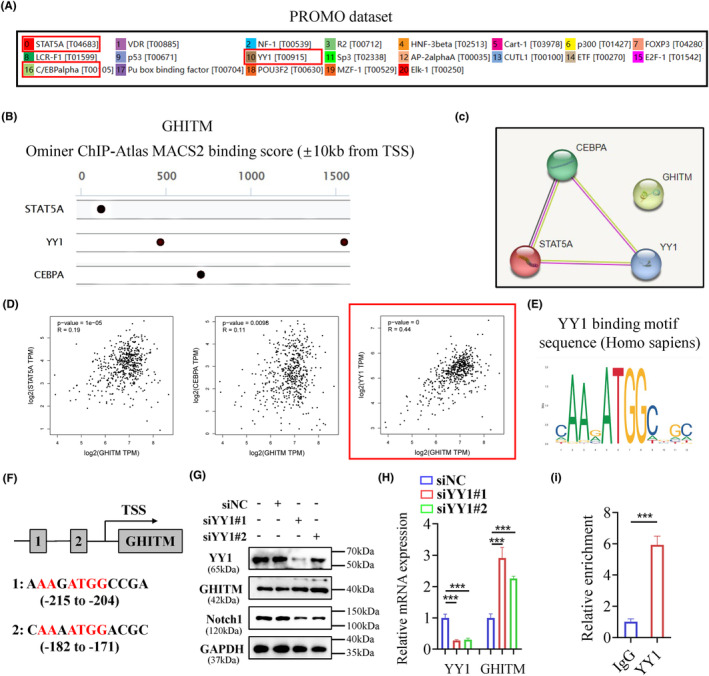
YY1 regulates the expression of GHITM via binding to its promoter in KIRC. (A) The PROMO web tool and (B) the Ominer web tool were used to predict the potential transcriptional factors of GHITM. (C) Using the STRING program to analyse the GHITM, STAT5A, YY1 and CEBPA. (D) Scatterplots of correlations between GHITM expression and the transcriptional factors we selected. (E) The YY1 binding motif sequence predicted by the JASPAR. (F) A diagram showed the sequence and position of the YY1 binding peak in the GHITM promoter. (G) Western blot and (H) qRT‐PCR analysis were used to explore GHITM and Notch1 expression after YY1 knockdown in 786‐O cells. (I) ChIP analysis of YY1 occupancy on GHITM promoter in 786‐O cells. ***p < 0.001.

**FIGURE 9:**
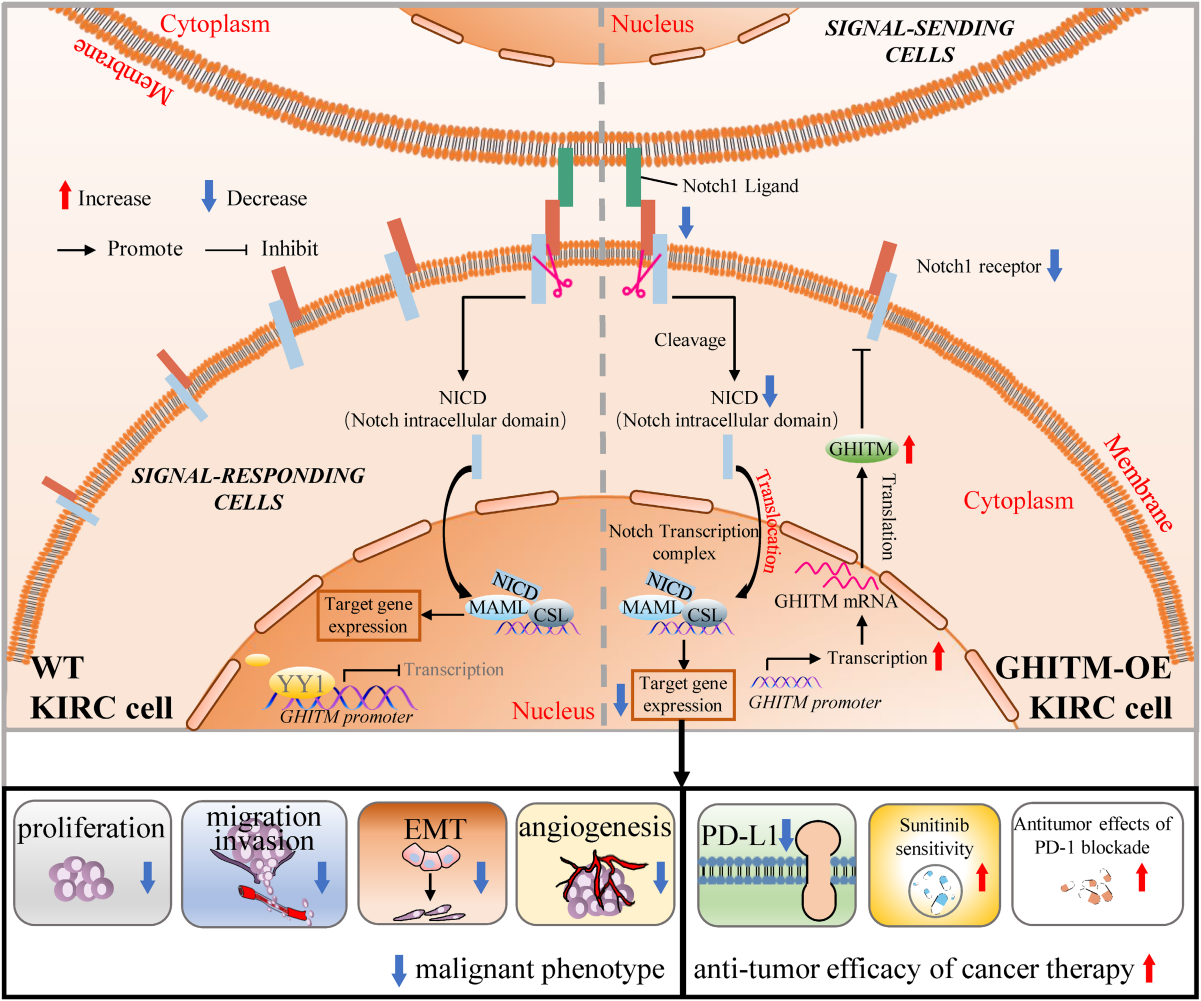
A model depicting that YY1 transcriptionally regulates the expression of GHITM, which modulates malignant phenotype and sensitivity to PD‐1 blockade of renal cancer cells via Notch signalling.

The following experiment was conducted to explore the regulatory mechanism of GHITM in KIRC cells. PROMO database and GeneCards database were used to predict the potential transcription factors of GHITM (Figure 8A,B). Among them, STAT5A, YY1 and CEBPA were found in both datasets (Figure 8C). The results of Pearson's correlation analysis showed there was a better correlation between GHITM and YY1 (Figure 8D), hence we next examined whether YY1 transcriptionally regulated the expression of GHITM in KIRC. The YY1 binding motif sequence predicted by the JASPAR database was mapped to predict promoter regions of GHITM, and two transcription factor‐binding sites were found (Figure 8E, F). We found that knockdown of YY1 increased the protein and mRNA levels of GHITM in 786‐O cell line (Figure 8G,H). Moreover, the ChIP–qPCR analysis indicated that YY1 was enriched in the promoter region of GHITM (Figure 8I). These results demonstrated that YY1 regulated GHITM gene transcription by binding to its gene promoter. Taken together, our data not only provide a molecular insight into YY1/GHITM/Notch1 axis but also reveal a promising therapeutic target for KIRC patients (Figure 9).

## 4 DISCUSSION

There are about 75% of newly diagnosed RCC patients that are KIRC, making it one of the most common genitourinary system malignancies.^1^ Globally, RCC incidence is increasing steadily, and 5‐year overall survival rate of early RCC is over 90% due to its organ‐confining.^32^, ^44^ Of note, once KIRC invades local organs or spreads distantly, the 5‐year survival rate of patient is just 10% even with advanced care.^45^, ^46^ The poor prognosis of patients with advanced stage are insensitive to the conventional radiotherapy and chemotherapy, the major treatment of KIRC is still surgical operation. In the current state of research, there is no efficient biomarker for prognosis prediction and early diagnosis of KIRC. Hence, it is of utmost importance to enhance our understanding of the mechanisms involved in KIRC tumorigenesis and discover a novel, sensitive and reliable biomarker which could serve as a promising prognostic indicator and therapeutic target for KIRC.

Little study is conducted to explore the biological role of GHITM in cancer, a majority of current research focuses on its effect on mitochondrial function.^33^ In this study, the analyses of the TCGA database and our own KIRC cohorts showed GHITM levels were downregulated in KIRC tissues. Downregulated GHITM was associated with clinicopathologic characteristics of KIRC and could predict poorer survival. Meanwhile, univariate and multivariate survival analyses revealed GHITM might be utilized as an independent prognostic factor for estimating KIRC patients' prognosis. Moreover, ROC curve revealed a high diagnostic value of GHITM in separating KIRC patients from healthy individuals, suggesting it might be a promising biomarker in the diagnosis of KIRC. Function experiments demonstrated that overexpressed GHITM could inhibit the malignant phenotype of KIRC cells. These results showed that GHITM might play an antioncogene role in KIRC progression.

Notch signalling could influence biological behaviour of cells by regulating of growth, differentiation and apoptosis.^12^ Notch1‐4 are single‐pass, the intracellular domain of Notch would be released after two proteolytic cleavages which caused by ligand binding, then it transits to the nucleus and exerts its biological functions.^47^, ^48^ Increasing evidence indicates dysregulation of Notch signalling pathway plays a significant role in various tumours tumorigenesis, including pancreatic carcinoma, melanoma, mammary carcinoma, glioma and so on.^49^ According to previous study, Notch inhibition perturbs growth of KIRC cells and the effect of Notch signalling on KIRC cells growth is specifically attributed to the Notch1. Exploration of LinkedOmics database and GSEA of the GSE126964 dataset prompted us GHITM may play its significance in cancer by regulating Notch signalling pathway. qRT‐PCR and Western blotting analysis showed that GHITM overexpression induced the downregulation of Notch1, and Notch1 overexpression could abolish the inhibitory effects of GHITM on malignant phenotype of KIRC cells. Thus, upregulation of GHITM may suppress tumorigenesis and progression of KIRC by inhibiting the Notch1 expression.

There has been much discussion about tumour‐associated immune cells and tumour immunotherapy in recent years, and tumour patients' prognosis is significantly influenced by infiltrating immune cells.^50^ Figure 6A showed that GHITM level was positively related to infiltration level of antitumor immune cells including B cell, CD4+ T cell and CD8 + T cell, and was negatively related to the infiltration level of cancer associated fibroblast cells, the protumor immune cells. Our findings elucidated GHITM could be an important regulator in cancer cells immunity and a new target for immunotherapy. Furthermore, expression of GHITM also correlated with the sensitivity of TCGA‐KIRC patients to chemotherapeutic drugs, which implied the possible significance of GHITM in personalized treatment of KIRC patients. Although many patients with advanced renal cell carcinoma who receive combination of an immune checkpoint inhibitor and a VEGF pathway inhibitor show better clinical benefit, most responsive patients would develop resistance over time.^51^, ^52^ Our study showed that ectopic overexpression of GHITM could enhance the sensitivity of renal cancer cells to sunitinib and the antitumor effects of PD‐1 blockade, which was consistent with previous bioinformatics analysis. However, the molecular mechanism underlying the effects of GHITM on the TILs and drug sensitivity needs further investigation.

Transcriptional control is an important way of regulating of gene expression. Hence, we explore the regulatory mechanism of GHITM in KIRC cells from the perspective of transcriptional level. The transcription factor, YY1, has been identified as a master regulator of many pathways involved in cell growth, survival, epithelial to mesenchymal transition (EMT), metastasis and so on.^53^ Li et al. reported that YY1/HDAC2 complex downregulated YTHDC1 to controls the sensitivity of renal cancer to sunitinib.^54^ Brenda et al. reported PTEN suppression of YY1 could induce HIF‐2 activity in VHL (von‐Hippel‐Lindau)‐null renal cell carcinoma.^55^ In this study, we observed that YY1 could regulate GHITM gene transcription by binding to its gene promoter, which revealed a novel YY1/GHITM/Notch1 axis in modulating the aggressive phenotypes of KIRC. Of note, the results presented in Figure 8G,H indicated that YY1 exerted inhibitory effect on GHITM expression in 786‐O cells, which contradicted the observed positive association between YY1 mRNA levels and GHITM mRNA levels in the TCGA‐KIRC cohort. Additionally, it is not yet understood whether GHITM could be regulated at the post‐transcriptional or post‐translational level in KIRC. Further studies are required to resolve these questions.

## 5 CONCLUSION

In conclusion, our study confirms the gene expression pattern and prognostic value of GHITM in KIRC, and provides evidence that GHITM inhibits KIRC cell proliferation, migration and invasion in vitro and in vivo by regulating Notch1 signalling. Importantly, GHITM could enhance the sensitivity of KIRC cells to sunitinib and potentiates the immunotherapy. Additionally, we further found that YY1 suppressed GHITM transcription via binding to its promoter directly. Based on these results, we propose that targeting the YY1/GHITM/Notch1 axis could decrease the malignant behaviour of KIRC cells and potentiate antitumour efficacy of cancer therapy, which provides a promising and novel therapeutic strategy for KIRC patients. However, further mechanism research is needed to elucidate whether there are additional downstream pathways involved in GHITM regulation of the malignant phenotype in KIRC, and whether YY1 requires the participation of the cotranscription factors to inhibit GHITM transcription.

## AUTHOR CONTRIBUTIONS

**Shiyu Huang:** Conceptualization (lead); data curation (lead); formal analysis (equal); software (equal); validation (equal); writing – original draft (lead). **Jiachen Liu:** Data curation (equal); investigation (equal); methodology (equal); resources (equal); supervision (equal); writing – original draft (equal). **Juncheng Hu:** Formal analysis (equal); funding acquisition (equal); project administration (equal); supervision (equal); validation (equal); writing – review and editing (equal). **Yanguang Hou:** Conceptualization (equal); methodology (equal); software (equal); visualization (equal). **Min Hu:** Data curation (equal); investigation (equal); software (equal); visualization (equal); writing – original draft (equal). **Banghua Zhang:** Conceptualization (equal); formal analysis (equal); investigation (equal); supervision (equal); writing – original draft (equal). **Hongbo Luo:** Funding acquisition (equal); supervision (equal); visualization (equal). **Shujie Fu:** Data curation (equal); investigation (equal); methodology (equal); software (equal); visualization (equal); writing – original draft (equal). **Yujie Chen:** Conceptualization (equal); formal analysis (equal); methodology (equal); software (equal). **Xiuheng Liu:** Funding acquisition (equal); investigation (equal); methodology (equal); supervision (equal); validation (equal). **Zhiyuan Chen:** Conceptualization (equal); formal analysis (equal); funding acquisition (equal); resources (equal); software (equal); validation (equal); writing – review and editing (equal). **Lei Wang:** Conceptualization (equal); funding acquisition (equal); methodology (equal); software (equal).

## CONFLICT OF INTEREST STATEMENT

The authors declare that they have no known competing financial interests or personal relationships that could have appeared to influence the work reported in this paper.

## Supporting information


Appendix S1.

## Data Availability

The data that support the findings of this study are available from the corresponding author upon reasonable request. The original contributions presented in the study are included in the article. Further inquiries can be directed to the corresponding authors.
