# Supplementary material for: GHITM regulates malignant phenotype and sensitivity to PD‐1 blockade of renal cancer cells via Notch signalling
Source: J Cell Mol Med. 2024 Apr 8;28(8):e18290. doi: 10.1111/jcmm.18290 (PMC11000813; doi:10.1111/jcmm.18290)
Supplement: Supplementary file 1 — Appendix S1. [file JCMM-28-e18290-s001.pdf]

Supporting Information for
ORIGINAL ARTICLE

GHITM regulates malignant phenotype and sensitivity to PD-1 blockade of renal cancer cells via Notch signaling

Shiyu Huang [1,2†], Jiachen Liu [1,2,3†], Juncheng Hu [1,2†], Yanguang Hou [1,2], Min Hu [4], Banghua Zhang[1,2,5], Hongbo Luo [1,6], Shujie Fu [1,2], Yujie Chen [1,2], Xiuheng Liu [1,2*], Zhiyuan Chen [1,2*], Lei Wang [1,2*]

[1] Department of Urology, Renmin Hospital of Wuhan University, Wuhan, 430060 Hubei, China

[2] Institute of Urologic Disease, Renmin Hospital of Wuhan University, Wuhan, 430060 Hubei, China

[3] Central Laboratory, Renmin Hospital of Wuhan University, Wuhan 430060, Hubei, China

[4] Department of Cardiology, Renmin Hospital of Wuhan University, Wuhan 430060, Hubei, China

[5] Hubei Key Laboratory of Digestive System Disease, Wuhan 430060, China.

[6] Department of Urology, The Second Hospital of Huangshi, Huangshi 435000, China

†These authors made equal contributions to this work.

*Corresponding author:

Xiuheng Liu, Zhiyuan Chen and **Lei Wang**

Tel/Fax: +86 027-88041911

E-mail: drliuxh@hotmail.com (Xiuheng Liu), chenzhiyuan163@163.com (Zhiyuan Chen) and drwanglei@whu.edu.cn (Lei Wang).

Extended detailed methods

ROS Assay and Flow Cytometry

For KIRC cell, a reactive oxygen species detection kit (Beyotime, Shanghai, China) was applied to assess its ROS level. Briefly, KIRC cells were stained with DCFH-DA in the dark at 37 °C for half an hour. After being washed three times with PBS, ROS level was detected by a fluorescence microscope or flow cytometry (Beckman Coulter Biotechnology, Suzhou, China).

Apoptosis of KIRC cells was analyzed with Annexin V-FITC/PI apoptosis kit (Beyotime, Shanghai, China) based on the instructions. KIRC cells with different pretreatments were collected and washed twice with PBS, thereafter, 195 μl binding buffer was added to resuspend cells. The cells were then stained with 10 μl PI and 5 μl Annexin V-FITC working solution for 15 min at room temperature in the dark. CytoFLEX (Beckman Coulter Biotechnology, China) was utilized for detection of apoptosis.

TdT-mediated dUTP Nick-End Labeling (TUNEL) Staining Analysis

A commercially available TUNEL kit (Beyotime, Shanghai, China) was utilized to detect apoptosis of KIRC cells. Briefly, KIRC cells (1×10^5 cells/well) were seeded into 24-well plates. The KIRC cells were fixed with 4% paraformaldehyde for 15 min and permeabilized with 0.2% Triton X-100 for 10 min before it was incubated with TUNEL staining reaction solution in the dark at 37°C for an hour. DAPI was used to visualize the nuclei and images were captured by the fluorescence microscope.

Supplementary Figures 1-6 and Figure Legends

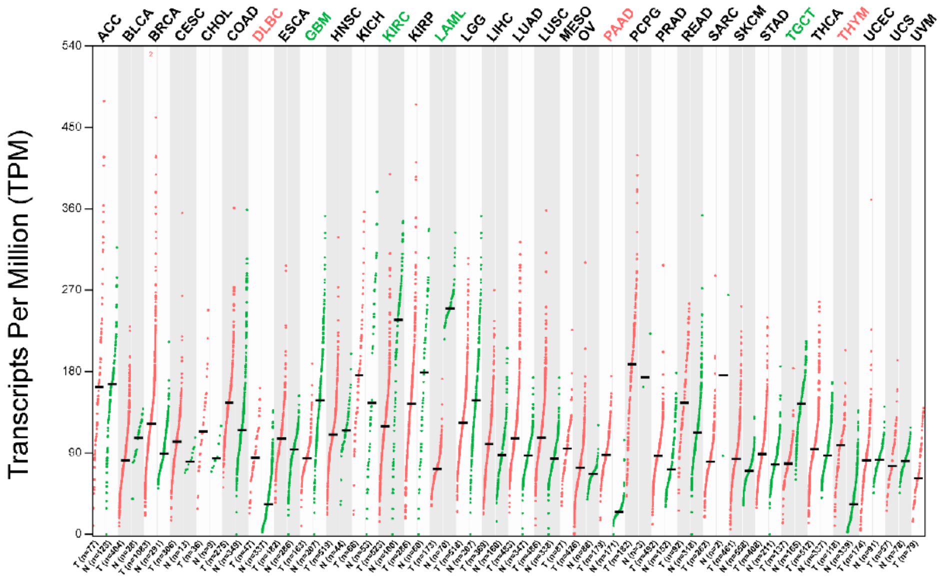

Figure S1 GHITM expression levels in different tumor types from TCGA and GTEx dataset were determined by GEPIA database.

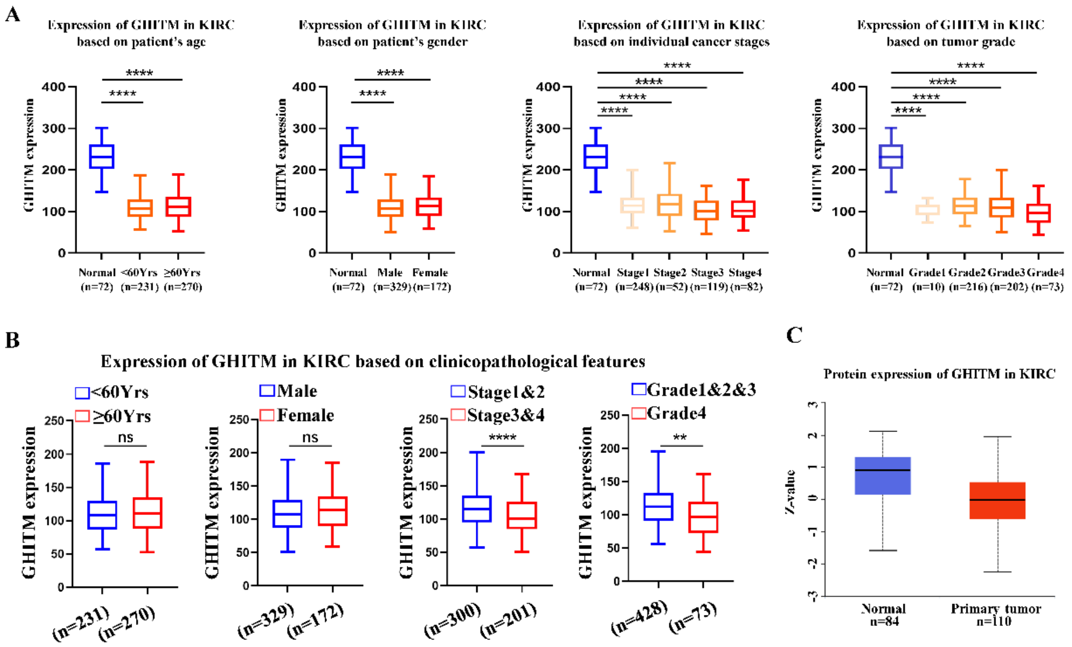

Figure S2 GHITM is downregulated in KIRC and correlated with clinicopathological features. (A) GHITM was downregulated in all the variables compared to the normal, including patient's age, patient's gender, individual cancer stages and tumor grade. (B) Expression analysis of GHITM

based on different variables including patient age, patient gender, individual cancer stages and tumor grade. (C) GHITM protein expression in KIRC analyzed by the UALCAN database. *p < 0.05; **p < 0.01; ***p < 0.001; ****p < 0.0001.

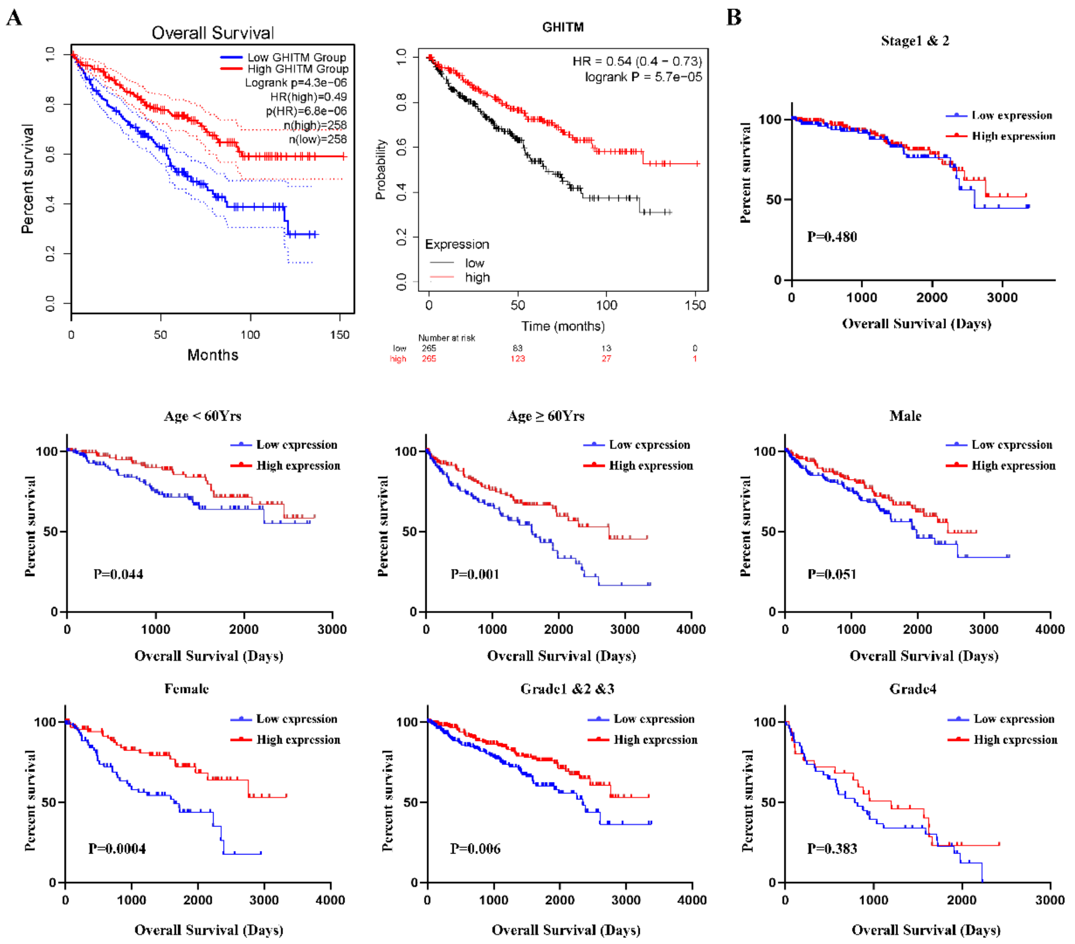

Figure S3 Diagnostic and prognostic value of GHITM in KIRC. (A) OS survival curves of KIRC patients in GEPIA database and Kaplan-Meier Plotter database. (B) Low expression of GHITM was associated with poor prognosis in most of subgroups.

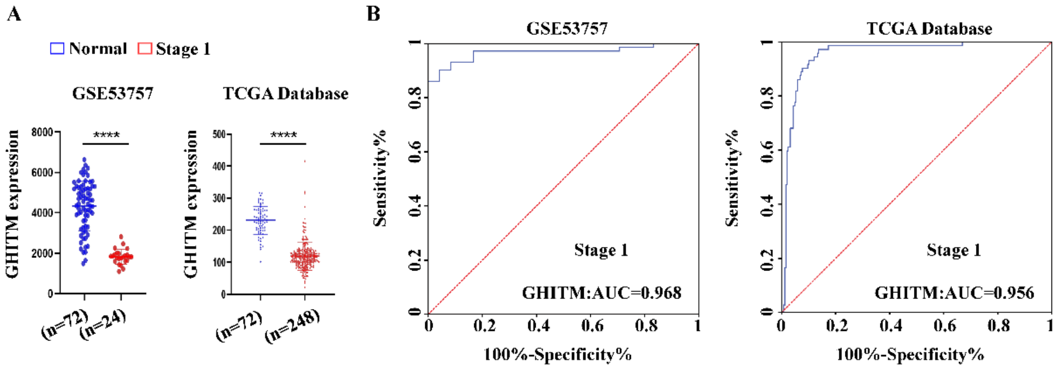

Figure S4 Diagnostic and prognostic value of GHITM in KIRC. (A) GHITM expression in normal tissues and stage I KIRC tumor tissues in GSE53757 and TCGA dataset. (B) ROC curve for stage I KIRC patients in GSE53757 and TCGA dataset. ****p < 0.0001.

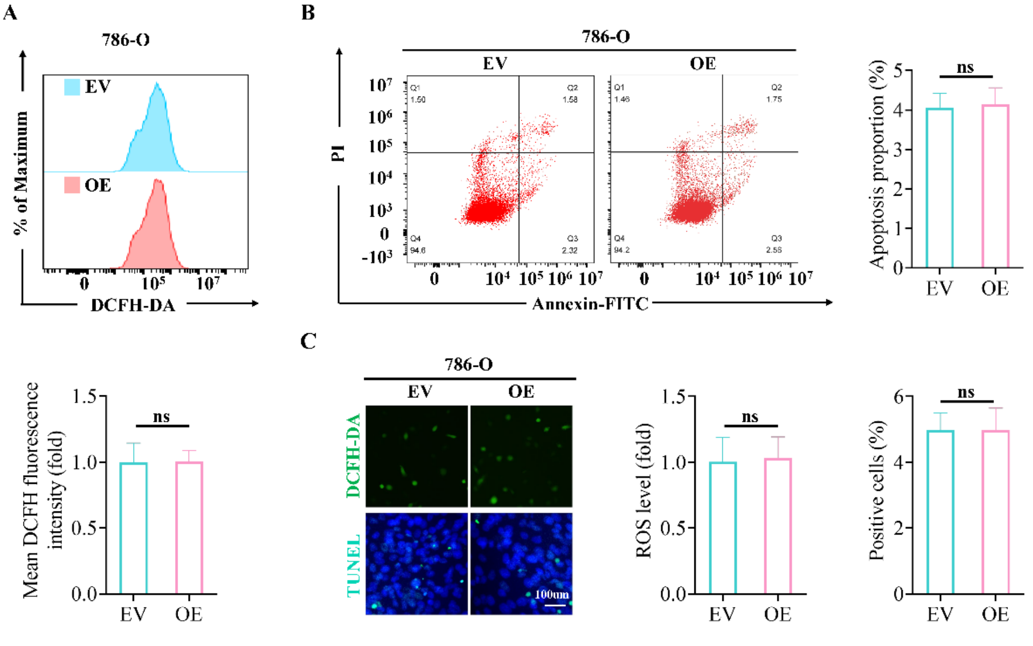

Figure S5 (A) ROS levels detected by flow cytometry. (B) Representative flow cytometry results for apoptosis in the indicated groups. (C) Representative DCFH-DA staining and TUNEL staining images and the statistical results.

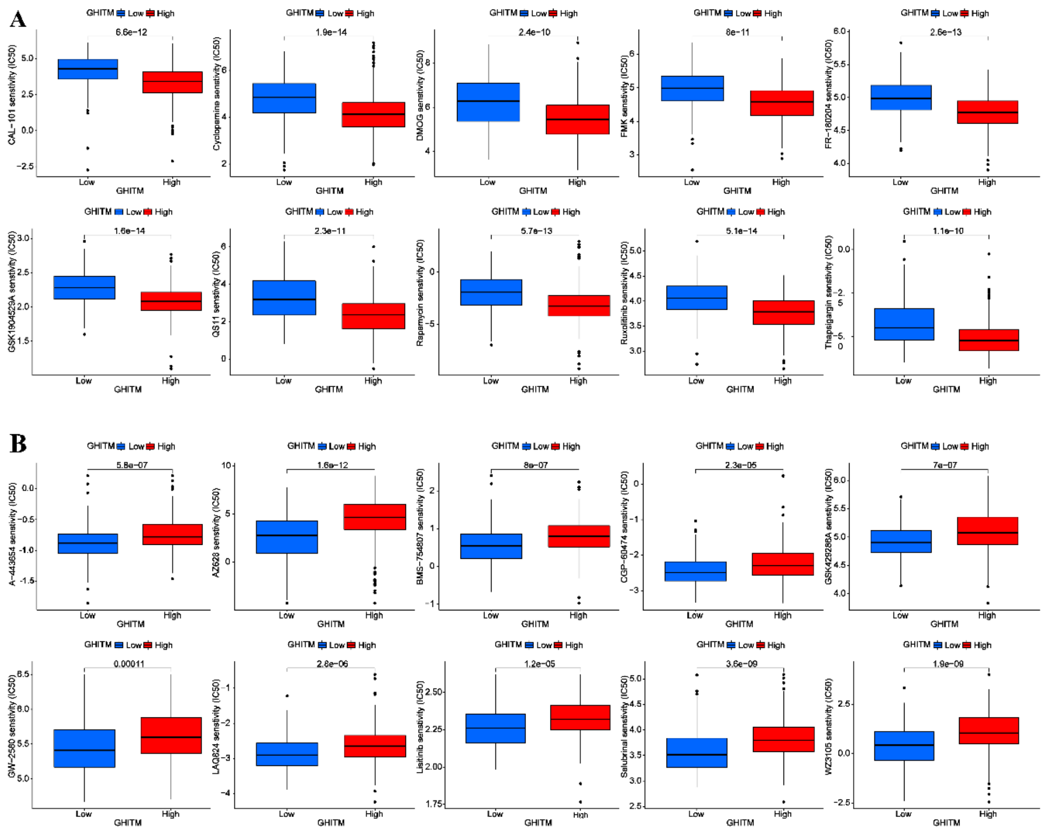

Figure S6 GHITM expression correlated with Tumor Immune Infiltrates (TILs) and drug sensitivity. The effect of GHITM on sensitivity of TCGA-KIRC patients to chemotherapeutic drugs which were validated to be effective in other kinds of cancer. (A) The drugs whose IC50 levels were higher in the GHITM low expression group. (B) The drugs whose IC50 levels were higher in the GHITM high expression group.

Supplementary Tables 1-4

Table S1 The sample information for indicated datasets.

Datasets.	Platform	Normal tissue	Tumor tissue	Total
GSE126964	GPL20795	11	55	66
GSE53757	GPL570	72	72	144
GSE40435	GPL10558	101	101	202

Table S2 Indicated primers used in PCR experiments

GENE	Primer sequences (5′-3′)
GHITM	F: ATTGGAACCATCGATGGAAAA
	R: ACAATGCTCCAAGACCAAC
GAPDH	F: ATCATCCCTGCCTCTACTGG
	R: GTCAGGTCCACCACTGACAC
Notch1	F: GGACCAGATTGGGGAGTT
	R: CACACTCGTCCACATCGT
Notch2	F: AAAAATGGGGCCAACCGAGAC
	R: TTCATCCAGAAGGCGCACAA
Notch3	F: GCAGCGATGGAATGGGTTTC
	R: CTGCCAGGTTGGTGCAGATA
Notch4	F: GCGGAGGCAGGGTCTCAACGGATG
	R: AGGAGGCGGGATCGGAATGT
PD-L1	F: TGGCATTTGCTGAACGCATTT
	R: TGCAGCCAGGTCTAATTGTTTT
YY1	F: GAAGCCCTTTCAGTGCACGTT
	R: ACATAGGGCCTGTCTCCGGTAT

Table S3 Association between GHITM level and the clinicopathological characteristics in TCGA-KIRC cohort.

Characteristic	No. of patients	GHITM level		Chi square value	p value
		low	high		
**Age (years)**				0.586	0.444
<60	231	120	111		
≥ 60	270	131	139		
**Gender**				2.365	0.124
Male	329	173	156		
Female	172	78	94		
**Stage**				12.500	**
1	248	109	139		
2	52	22	30		
3	119	72	47		
4	82	48	34		
**Grade**				9.104	*
1	10	4	6		
2	216	99	117		
3	202	100	102		
4	73	48	25		

*$p < 0.05$; **$p < 0.01$.

Table S4 Correlation between GHITM expression and the clinicopathological features of KIRC patients in GSE40435.

Characteristics	No. of patients	GHITM expression		Chi square value	p-value
		Low	High		
**Age (years)**				4.733	*
<60	31	10	21		
⩾ 60	70	39	31		
**Gender**				1.860	0.173
Male	59	32	27		
Female	42	17	25		
**Tumor Grade**				11.411	**
1	22	9	13		
2	47	17	30		
3	24	16	8		
4	8	7	1		

*$p < 0.05$; **$p < 0.01$.
